# Supplementary material for: Characterization of Small Extracellular Vesicles Isolated from Aurelia aurita
Source: Biology (Basel). 2025 Jul 23;14(8):922. doi: 10.3390/biology14080922 (PMC12384044; doi:10.3390/biology14080922)
Supplement: Supplementary file 1 [file biology-14-00922-s001.zip › biology-3734668-supplementary.pdf]

Supplementary Materials

Characterization of Small Extracellular Vesicles Isolated from *Aurelia aurita*

Aldona Dobrzycka-Krahel [1], Aleksandra Steć [2], Grzegorz S. Czyrski [3], Andrea Heinz [3] and Szymon Dziomba [2,*]

[1] Business Faculty, WSB Merito University in Gdansk, Al. Grunwaldzka 238 A, 80-266 Gdansk, Poland; aldona.dobrzycka-krahel@gdansk.merito.pl

[2] Department of Toxicology, Faculty of Pharmacy, Medical University of Gdansk, 107 Hallera Street, 80-416 Gdansk, Poland; aleksandra.stec@gumed.edu.pl

[3] LEO Foundation Center for Cutaneous Drug Delivery, Department of Pharmacy, University of Copenhagen, 2100 Copenhagen, Denmark; grzegorz.czyrski@sund.ku.dk (G.S.C.); andrea.heinz@sund.ku.dk (A.H.)

* Correspondence: szymon.dziomba@gumed.edu.pl

Zeta potential measurements with CE

The Zeta potential of EVs was calculated based on the CE experiments using the following equations:

$$V = \mu E \quad (1)$$

where V is the migration velocity of the solute, μ is the electrophoretic mobility of the solute, and E is the electric field strength;

$$\mu_{app} = \mu_{EOF} + \mu_{eff} \quad (2)$$

where μ_{app} and μ_{eff} are the apparent (observed) and effective electrophoretic mobility of the analyte, respectively, and μ_{EOF} is the electrophoretic mobility of the electroosmotic flow (EOF);

$$\mu = \frac{\varepsilon_r \varepsilon_o \zeta}{\eta} \quad (3)$$

where ε_r and ε_o are the relative electric permittivity of the electrolyte and vacuum, respectively, η is the dynamic viscosity, and ζ is the ζ-potential.

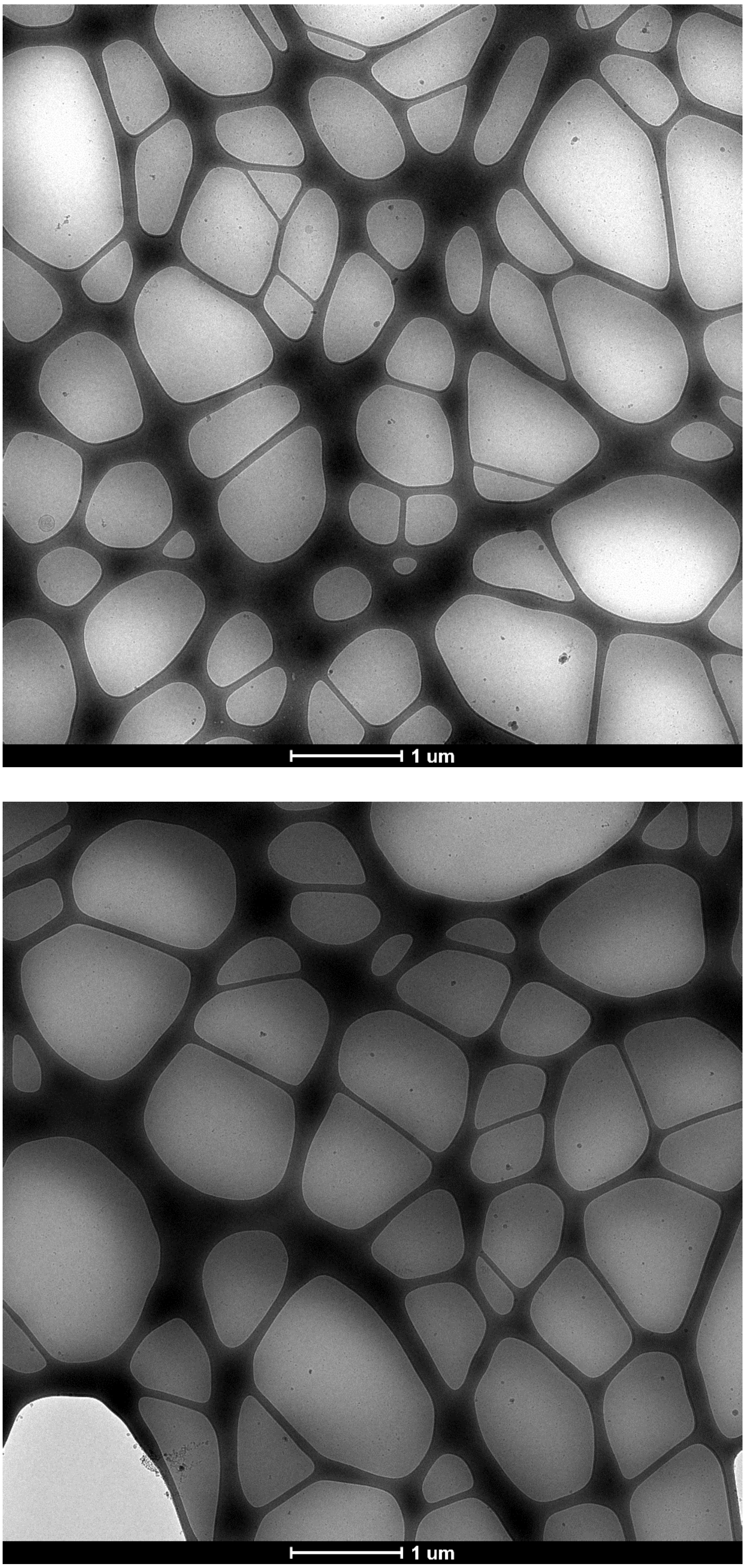

Figure S1. A zoom-out cryo-TEM images of EVs in Figure 1B.
